# Stress, glucocorticoid receptors, and adult neurogenesis: a balance between excitation and inhibition?

**DOI:** 10.1007/s00018-014-1568-5

**Published:** 2014-02-13

**Authors:** Dirk-Jan Saaltink, Erno Vreugdenhil

**Affiliations:** 1 Department of Medical Pharmacology, Leiden University Medical Center/Leiden Amsterdam Center for Drug Research, 2300 RC Leiden, The Netherlands; 2 Laboratory of Neurophysiology, Department of Molecular Cell Biology, Leiden University Medical Center (LUMC), Einthovenweg 20, 2333 ZC Leiden, The Netherlands

**Keywords:** Hippocampus, Post-mitotic maturation, Depression, Neural stem cell, Dentate gyrus, MicroRNA, DCX, Glutamate

## Abstract

Adult neurogenesis, the birth of new neurons in the mature brain, has attracted considerable attention in the last decade. One of the earliest identified and most profound factors that affect adult neurogenesis both positively and negatively is stress. Here, we review the complex interplay between stress and adult neurogenesis. In particular, we review the role of the glucocorticoid receptor, the main mediator of the stress response in the proliferation, differentiation, migration, and functional integration of newborn neurons in the hippocampus. We review a multitude of mechanisms regulating glucocorticoid receptor activity in relationship to adult neurogenesis. We postulate a novel concept in which the level of glucocorticoid receptor expression directly regulates the excitation-inhibition balance, which is key for proper neurogenesis. We furthermore argue that an excitation-inhibition dis-balance may underlie aberrant functional integration of newborn neurons that is associated with psychiatric and paroxysmal brain disorders.

## Background

“In the adult centres, the nerve paths are something fixed, ended, and immutable. Everything may die, nothing may be regenerated.” These words from Ramon y Cajal in 1913 [1] highlight what has been one of the central dogma’s in neuroscience for a long time: the birth of new neurons, i.e., neurogenesis, was restricted to prenatal and early postnatal development and that the adult mammalian brain was unable to produce new neurons. However, in the 1960s, Joseph Altman and colleagues showed first evidence for adult neurogenesis in the brain of rodents [2–4]. Although these data were received with skepticism by the scientific community, results were reproduced and revealed the neuronal phenotype derived from dividing cells in the hippocampus [5].

In the 1990s, pioneering studies by Weiss and colleagues identified stem cell-like cells from the adult brain that were able to differentiate into neurons and astrocytes [6]. Important for the acceptance of the concept of adult neurogenesis has been the development of novel techniques and scientific methods. Cell division for example, can be marked using bromodeoxyuridine (BrdU) or [^3^H]thymidine. These molecules label and incorporate into DNA of dividing cells and can be visualized with electron and confocal microscopy [7]. By varying the paradigm and the examination time points after injection, these techniques allow quantitative analysis of proliferation, differentiation, and survival. During the last decades, it also became clear that developing neurons express distinct markers during their maturation process [8]. BrdU-labeled DNA in combination with immuno-histochemical analysis of the expression of these specific neuronal markers by confocal microscopy unambiguously revealed the existence of neurogenesis in the adult brain [9]. For example, for immature newborn neurons, doublecortin (DCX) is regularly used, while for mature neurons the specific adult neuronal marker of nuclei NeuN is mostly used [10–12]. Analysis of adult born neurons can also be performed using retroviral genetic marking, since retroviruses exclusively enter the target cell during mitosis [13]. Adult neurogenesis has been shown in the brain of different species of rodents [14, 15], primates [16], and even humans [17, 18]. Newborn neurons have been observed to integrate into neuronal hippocampal circuits and have been functionally associated with complex brain processes like cognition, emotion, and pattern separation.

The discovery of environmental factors regulating adult neurogenesis in a bi-directional manner has been of key importance for the acceptance of the concept [19]. In particular, the finding that stress and adrenal steroid hormones down-regulate adult neurogenesis [14, 20] contributed significantly. As aberrant forms of stress and chronic elevation of adrenal hormones are also linked to the psychopathology of depression, possible involvement of adult neurogenesis in psychiatric diseases has been a topic of intense research. Nowadays, changing rates of adult neurogenesis have been linked to aging, environment, hormones, neurochemicals, and behavior as well as to numerous brain diseases ranging from depression to epilepsy.

Overthrowing a dogma always attracts attention. Therefore, an impressive number of papers have appeared in the last decade and as a consequence, numerous reviews have been published covering specific topics of adult neurogenesis such as general mechanisms and signaling cascades [13, 21], cognition and memory formation [22–24], evolution [25], olfaction [26] psychiatric diseases [27], paroxysmal disorders such as epilepsy [28, 29], and neurodegenerative disorders [30]. Also, the concept of stress and its effect on adult neurogenesis have been reviewed extensively [31–35]. Therefore, we will only briefly review the general aspects of the stress system and the interplay between stress and adult neurogenesis in the hippocampus. More extensively, we will highlight novel findings on the function of the glucocorticoid receptor, the main mediator of the stress response, in neuronal progenitor cells and in adult neurogenesis. We will present a novel concept about the role of the glucocorticoid receptor in the positioning and functional integration of newborn neurons and we discuss how this concept may contribute to the chronification of paroxysmal brain diseases.

## Neurogenesis in the adult hippocampus

**Fig. 1:**
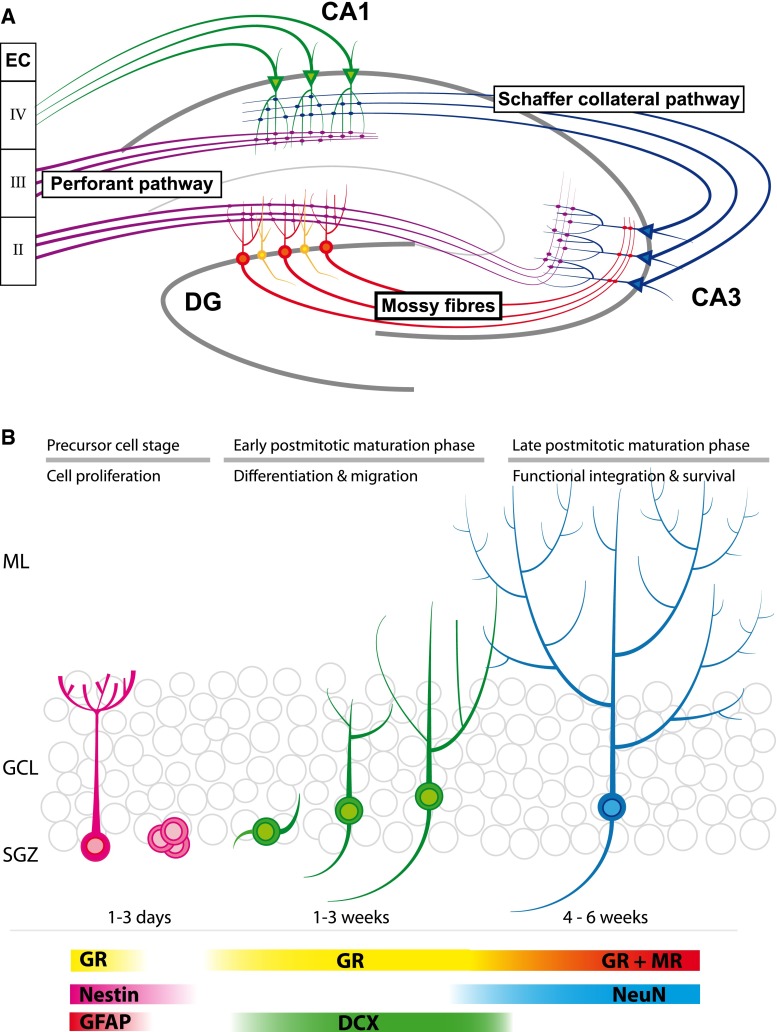
Overview of the hippocampal formation and adult neurogenesis. **a** Schematic representation of the tri-synaptic pathway. Axons derived from the entorhinal cortex (EC) layer II projects via the perforant pathway (*purple*) towards the dendrites of the DG granule cells (*red*). The perforant pathway projects also towards CA3 were they end in boutons, which contact dendrites of pyramidal cells (*blue*). Mossy fibers (*red*) originating in the DG granule cells project to the same pyramidal cells in the CA3. Via the Schaffer collateral pathway (*blue*) the CA3 projects towards pyramidal neurons (*green*) in the CA1, which also receive input from the EC layer III. CA1 pyramidal neurons project (*green*) towards layer IV of the same EC. Only in the DG, new-born granule cells (*yellow*) integrate into this network. **b** Adult neurogenesis can be divided in three main stages: the precursor cell stage, the early postmitotic maturation phase and the late mitotic maturation phase. Stem cells proliferate in the sub-granular zone (SGZ) where after NPCs migrate into the granular cell layer (GCL). During the late postmitotic phase, the newborn neurons develop dendritic trees protruding into the molecular layer (ML). Different stages are characterized by expression of specific markers. Note that the GR is expressed in radial glia cells, but not early precursor cells. For further details, see main text

To understand the possible relevance of adult neurogenesis, it is important to understand the anatomy of the hippocampus. The hippocampus can be divided into three main subregions, i.e., CA1, CA3, and dentate gyrus (DG). Neuronal cells derived from these three subregions are connected by so-called trisynaptic pathways (see Fig. 1a). It is generally assumed—but not proven—that information processing by this tri-synaptic circuit is crucially involved in learning and memory formation. Neurogenesis occurs only in the DG and there is no evidence that other hippocampal regions generate new neurons [36].

The DG area consists of three layers: the molecular layer (ML), the granule cell layer (GCL), and the hilus or polymorphic layer (PL; see Fig. 1b). The GCL is densely packed and consists of a stack of roughly six granule cell bodies [2, 3, 37]. These cells have an elliptical cell body of about 10–18 μm, are tightly packed, and are not ensheathed by glia cells. Granule cells have a cone-shaped dendritic tree that projects into the ML. At the other site of the cell body, granule cells give rise to unmyelinated axons called mossy fibers. These fibers have large boutons, which not only connect to pyramidal cells of the CA3 but also contact mossy cells in the hilus [2, 3, 38]. The border between GCL and hilus is called the subgranular zone (SGZ) and inhabits neural stem cells (NSCs), which are the source of adult neurogenesis and generate excitatory granule cells [5, 36].

Neurogenesis in the adult DG can be divided into three main stages (see Fig. 1b) [7, 36]. The first stage is called the precursor cell stage and is characterized by cell division [6, 39]. The primary NSC in the DG is a radial glia (RG)-like astrocyte called the type 1 cell [9, 36]. Like RGs, these NSCs have long processes that project into the ML. The second phase is called the early postmitotic phase and is characterized by neuronal differentiation and migration of neuroblasts/immature neurons. Under the influence of GABA, they start to develop dendrites and axons towards, respectively, the ML and CA3 regions. At this stage, the dendrites lack any spines but receive functional GABAergic input [13, 21, 40]. Initially, GABA has an excitatory effect, which induces dendritic growth. When this excitatory signaling is blocked, immature neurons develop abnormally [22–24, 41].

The immature neurons/neuroblasts migrate radially into the DG. Doublecortin, a gene that is causally involved in migration of neuronal precursor cells and neuroblasts [42], is a major marker in this process (see Fig. 1b). The majority of newborn cells end up in the inner third layer of the DG, while 10–20 % reach the mid third layer and only a few cells ends up in the outer third layer [37, 40]. This non-proportional distribution may be explained by the fact that the majority of GCs, which inhabit the middle and outer third layers, are born during early postnatal development while the inner third layer is generated later during development [43].

The third stage is called the late postmitotic maturation stage, wherein surviving cells integrate into the local DG network and further mature into genuine granule cells [40, 44–46]. Although newborn cells lose the expression of immature markers around 4–5 weeks after cell birth, full maturity is reached around 60 days after cell birth [44]. After the first weeks, newborn neurons continue to extend their dendritic and axonal processes and many new connections are formed with glutamatergic synapses from the EC and output to pyramidal cells in the CA3. Not only newborn GCs increase and reshape their synaptic connectivity. Due to their interaction with newborn GCs, mature GCs also change their connectivity. Spines from newborns’ GCs compete at entorhinal boutons with old synapses from mature GCs and—possibly due to local glutamate spillover at the synapse—will replace the old synaptic connection [47–49]. This pattern of competitive synaptic plasticity in the molecular layer also seems to be present in the hilus and CA3 region at the axonal end of newborn neurons. Although boutons actively connect to synapses already 17 days after cell division, the connections are fully mature 2 months post-cell division [47, 50].

## The stress system, glucocorticoids, and glucocorticoid receptors

Stress is the response of an organism aiming to maintain a physiological balance called homeostasis. When homeostasis is challenged by a stressor, the organism responds by behavioral and physiological adaptations, resulting in coping and recovery. A stressor can be an environmental challenge, e.g., taking a test, or a physical challenge, e.g., a viral infection. Whatever the nature of the stressor, the body reacts in a stereotypical way [51]. Firstly, neurohormones, in particular corticotropin-releasing hormone (CRH), are released in the hypothalamus that subsequently project to the pituitary [52]. Secondly, activation of CRH receptor expressing neuro-endocrine cells in the pituitary leads to the subsequent release of adrenocorticotropic hormone (ACTH) into the blood circulation, which activates endocrine cells in the adrenal cortex. Thirdly, ACTH stimulates the release of adrenal glucocorticoid hormones, also called stress hormones, which are cortisol in man and corticosterone in rodents, here collectively referred to as CORT. This adrenal steroid hormone affects many organs in the body: it causes release of glucose in the blood circulation, it acts as a repressor of the immune system, and in the brain it facilitates information processing in limbic neuronal networks involved in emotion, cognition, and memory formation [for a review, see 31, 32]. CORT also functions as a feedback mechanism to the same brain structures in the hypothalamus that triggered its release, thereby stabilizing the hypothalamus–pituitary–adrenal (HPA) axis and preventing sensitive organs in the body from CORT overexposure. HPA axis activity is organized in a circadian rhythm with high levels in the morning, enabling individuals to cope with energy demands ahead of them. It is important to note that rapid HPA axis activation by *acute* stress and the subsequent turn-off of the HPA axis by the negative feedback response of CORT is healthy, as it helps an individual to cope with the stressor. However, dysregulation of the HPA axis by e.g., *chronic* stress may endanger the immune system, cardiovascular functions, the regulation of fear, cognition, and memory formation, and as such is associated with numerous diseases, in the brain in particularly with depression [33, 53].

Lipophilic CORT passes the blood–brain barrier easily and enters neuronal target cells by penetrating across the cell membrane. At the neuronal level, CORT controls the stress response through binding to two types of steroid receptors in the cytosol: the mineralocorticoid receptor (MR or NR3C2; [54]) and the glucocorticoid receptor (GR or NR3C1; [55–57]). These steroid receptors belong to a superfamily of ligand-inducible, highly conserved nuclear hormone receptors. Upon binding CORT, GR and MR translocate to the nucleus where they affect expression of specific sets of genes in two ways: (1) by binding to specific so-called glucocorticoid-response element DNA motifs located in promoter regions of target genes; a process called transactivation and (2) by protein–protein interaction with other transcription factors and co-factors, such as cAMP-response element binding (CREB) protein and CREB binding protein (CBP), thereby mainly inhibiting the activation of these transcription factors and as such is called transrepression (for review, see [58]). In addition, CORT evokes fast non-genomic neuronal responses by binding to membrane-bound GR and MR [59]. As these membrane-bound receptors have not yet been identified in NPCs, they are outside the scope of this review.

The hippocampus, a brain area crucially involved in cognition and memory formation, expresses both MR and GR at high levels, and is therefore particularly sensitive for fluctuating levels of CORT. Moreover, as neuronal stem cells in the dentate gyrus of the hippocampus are located in the close vicinity of blood vessels [60], stress and stress-induced elevated CORT levels may target NPCs easily and may be very profound (environmental) factors affecting adult hippocampal neurogenesis.

## The glucocorticoid receptor and adult neurogenesis

**Fig. 2:**
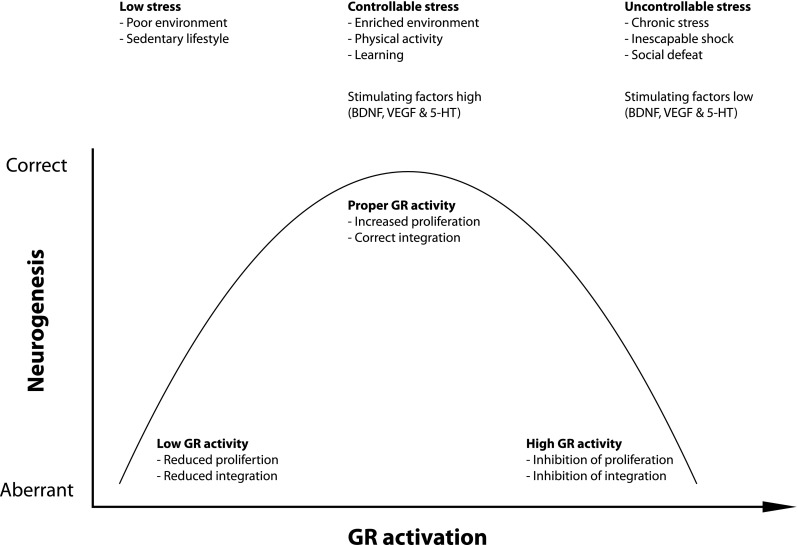
Relationship between GR activity and neurogenesis GR activation exerts both positive and negative effects on neurogenesis. We propose an inverted U-shaped model in which there is a relationship between the amount of GR activation and neurogenesis. Low levels of stress seen in animals kept in a poor environment or with a sedentary lifestyle induce low levels of proliferation and maturation. Controllable stress, like enriched environments, physical activity, and learning, coincides with increased levels of GR activation and is associated with increased cell proliferation and correct integration of mature neurons. Too much GR activation as seen during uncontrollable stress negatively affects proliferation and neuronal integration. Neurogenesis-controlling molecular factors, like BDNF, VEGF, and 5-HT signaling, are regulated by low–high GR activity in opposite directions

The general view is that stress and stress hormones inhibit adult neurogenesis by inhibiting proliferation of type 2 cells. For example, several chronic stress paradigms, like subordination stress in primates and social defeat in rodents showed diminished cell proliferation in the DG [16, 61–63]. Also, ground-breaking studies in the early 1990s showed that administration of adrenal hormones in rats negatively affect the incorporation of 3H-thymidine while removal of adrenal hormones by adrenalectomy booster the appearance of 3H-thymidine labeled cells [14, 64, 65], suggesting an inhibitory role for stress-induced glucocorticoids in adult neurogenesis. However, the relationship between stress and adult neurogenesis seems far more complex than a simple inhibitory role. For example, the use of the running wheel by mice is well known to booster adult neurogenesis at the proliferation stage and promotes neuronal differentiation [45, 66, 67]. At the same time, physical exercise is a strong activator of the HPA axis, leading to elevated levels of circulating glucocorticoids [68, 69]. Similarly, an enriched environment stimulates neuronal differentiation and survival of newborn cells [66, 70, 71], yet it simultaneously increases glucocorticoid levels [72]. Also, several learning paradigms not only stimulate survival of newborn neurons but also increase HPA axis activity and glucocorticoid levels [73, 74]. Recently, acute stress, induced by 3 h of immobilization stress resulting in elevated plasma CORT levels, was shown to induce (not repress) cell proliferation in the DG [75]. Reversely, hippocampal neurogenesis may also facilitate normalization of glucocorticoid levels after stress [76], suggesting a bi-directional relationship between adult hippocampal neurogenesis and regulation of the HPA axis. Clearly, the effect of stress and stress hormones on adult neurogenesis is complex. These paradoxical findings may be explained by several factors (see also Fig. 2). Firstly, neuronal stem cells in the SGZ of the dentate gyrus are located in a specialized microenvironment, the so-called neurogenic niche, consisting of numerous different cell types, including astrocytes, ependymal cells, blood vessels, interneurons, oligodendrocytes, and myeloid cells, i.e., microglia cells and dendritic cells. All of these cell types may modulate adult neurogenesis. For example, depending on the type of microglia and on the challenge, activated microglia cells release cytokines that may have detrimental or beneficial effects on adult neurogenesis (for review, see [77]). Also, these cell types express GRs indicating that stress-induced glucocorticoid elevation targets these cells as well, and as such these cells may modulate the rate of neurogenesis. Secondly, the nature of the stressor is an important factor. Control or no control over stress may have opposite effects on neuronal plasticity including adult neurogenesis whereby non-controllable stress such as social defeat or learned helplessness has a negative and controllable stress such as voluntary exercise or enriched environment has a positive effect on neurogenesis [72]. The precise neurochemical mechanisms that are differentially activated by controllable and uncontrollable stress are presently unknown but may involve desensitized serotonergic signaling via the dorsal raphe nucleus [78] as serotonin stimulates adult neurogenesis through 5HT1A receptors [79]. Thirdly, the duration of stress, acute stress (e.g., during learning) versus chronic stress, may have opposite effects on adult neurogenesis. Chronic stress has been associated with decreased expression of 5-HT1A receptor expression [80] and thus likely impaired serotonergic signaling in dentate gyrus region of the rodent hippocampus [81, for review see 82] while an enriched environment stimulates 5HT1A expression [83]. Fourthly, early life experiences may also affect the rate of neurogenesis in adult life. For example, exposure to* E. coli* bacteria in early life not only affects the responsiveness of microglia in the adult brain but also negatively affects the rate of adult neurogenesis after infection compared to non-*E. coli*-treated pups [84].

Besides serotonergic signaling, controllable versus non-controllable stress and acute versus chronic stress may also affect other transmitter systems. Relevant for adult neurogenesis particularly is the interaction with glutamatergic signaling, e.g., acute cortisol exposure may facilitate clustering of AMPA receptors [85], thereby facilitating the pro-plasticity activities of glutamate in the early and post-mitotic maturation phase. Another important group that interacts with stress and stress hormones is neurotrophins, like BDNF, VEGF, and IGF-1 and their Trks. Expression of these neurotrophins is not only regulated by stress and circulating stress hormones (see e.g., [86]) but also by an enriched environment, chronic stress, and by learning, and may be differentially regulated by controllable versus non-controllable stress [87]. The interplay between the stress system on the one hand and neurotrophins and excitatory stimuli on the other may direct the outcome on the proliferation, maturation, and functional integration of newborn cells in the DG. The nature of the stress, e.g., acute versus chronic, and the background of an organism—aging, traumatic early life events, environmental enrichment—further determine the outcome on adult neurogenesis. For more in-depth reviews on this topic, we refer to recent excellent papers highlighting the complex interplay between stress, stress hormones, and adult neurogenesis [27, 34, 39, 88–90].

## The glucocorticoid receptor

Corticosterone has profound effects on the excitation-inhibition balance within the hippocampus. This effect is not identical in all hippocampal sub areas. In both CA1 and CA3, high concentrations of corticosterone enhance LTP via short-term MR activation. After this acute effect, GR-mediated inhibition of the LTP normalizes the excitation-inhibition balance in these brain areas. However, within the dentate gyrus, the effect of corticosterone on the excitation-inhibition balance seems reversed. Despite the abundant expression of the GR within the DG, high concentrations of corticosterone do not suppress LTP in the long run but, in contrast to the CA1 and CA3, enhance LTP [91].

The MR and the GR are the main intracellular mediators of the stress response (for review, see [31–33]). In the DG, both receptors are abundantly expressed in all GCLs except in the SGZ. In neuronal precursor cells, MR expression is below detection levels and the GR is expressed in about 50 % of neuronal precursor cells, both in vivo [65, 92] as well as in primary neuronal precursor cell cultures [93]. These data suggest that the GR (and not the MR) is the main mediator of direct stress-suppressive effects on cell proliferation. Indeed, pharmacological blockade of the GR prevents the negative effect of exogenous CORT administration on the proliferation of neuronal progenitor cells [94]. The absence of both MR and GR expression in a subpopulation of neuronal progenitor cells opens up the possibility that the effect of stress hormones is indirect, likely via NMDA receptors. Activation of NMDA receptors inhibits and NMDAR blockade promotes cell proliferation in the dentate gyrus [95–97]. In addition, stress and CORT are known to stimulate glutamate release [85, 98]. Therefore, it may well be possible that the CORT-suppressive effects are mediated by glutamatergic signaling. Indeed, pharmacological blockade of NMDAR by MK801 prevents the inhibitory effect on cell proliferation by CORT administration [99], suggesting that glutamate-induced inhibition acts downstream of CORT signaling.

**Fig. 3:**
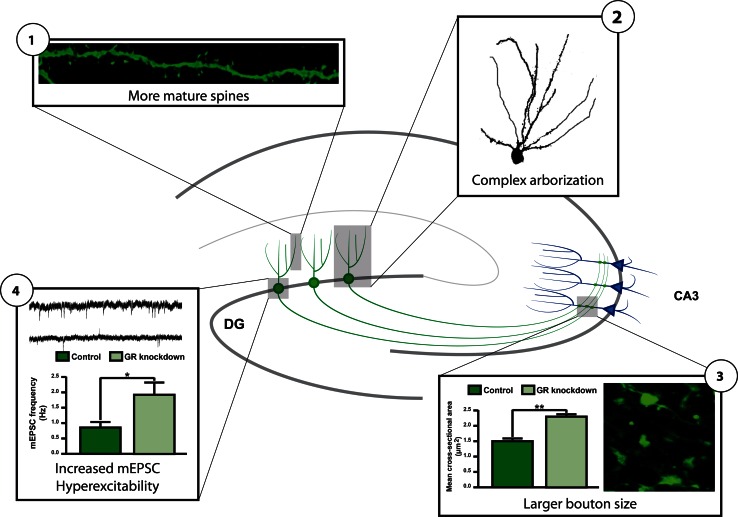
GR knockdown stimulates differentiation of post-mitotic neuronal precursor cells. GR knockdown increases the number of dendritic mature mushroom spines (*1*), axonal bouton size (*2*), and number of mini-excitatory postsynaptic currents (mEPSC; *4*). GR knockdown also leads to more complex dendritic arbors (*2*) and ectopically located new-born granule cells (not illustrated). After [108]

The presence of GR expression in a number of progenitor cells and in immature neurons [92] also suggests a direct effect of circulating CORT on adult neurogenesis. Such a direct role for the GR is further suggested by the fact that GR activity and GR mRNA and GR protein levels are tightly regulated by a number of factors that also regulate adult neurogenesis (see Table 1). One of these factors is doublecortin-like (DCL), a protein that is specifically expressed in radial glia cells during embryonic neuronal development [100] and that is co-expressed with DCX in progenitor cells in the adult hippocampus [101]. DCL belongs to the DCX gene family [102] and is a microtubule-associated protein regulating retrograde transport of activated GR proteins to the nucleus, indicating that progenitor cells possess specialized mechanisms to quickly translocate activated GRs. Another factor is microRNA(miR)-124, a small non-coding RNA that is able to bind to the 3′-untranslated region of the GR mRNA thereby reducing GR protein levels to 70 % [103]. Interestingly, miR-124 is a neuron-specific microRNA that directs progenitor cells in the brain to differentiate into a neuronal phenotype [104, 105]. To achieve neuronal fate, miR-124 represses the activity of a number of proteins such as REST [106] and Sox9 [105] that are known to antagonize neuronal differentiation. The repression of the GR by miR-124 suggests that reduced GR protein levels are critical for proper neuronal differentiation. Indeed, downregulation of GR proteins by retroviral and lentiviral delivery of GR-targeting small interference (si)RNA molecules specifically in neuronal progenitor cells in the mouse DG [107] accelerates their neuronal differentiation. Moreover, newborn granule cells with reduced GR protein levels exhibit more complex dendritic arbors, have increased numbers of mature dendritic spines, and more mature mossy fiber boutons. In line with this, cells with reduced GR expression exhibit increased basal excitability (see Fig. 3) [108]. A striking finding in this study was the positioning of newborn granule cells in the GCL: a large percentage of the cells with reduced GR levels were located in the middle and outer layer. This position is significantly different from newborn granule cells with normal GR expression that were predominantly present in the inner and middle layer. These data suggest a role for the GR in the accurate migration and functional integration of newborn cells in the GCL, a role which is in line with the effect of glucocorticoids on cortical neuron migration in embryonic development [109] and the changed positioning of DCX-positive newborn granule cells after adrenalectomy [108].

**Table 1:** Overview of factors affecting glucocorticoid receptor activity and adult neurogenesis

	GR			Adult neurogenesis		
	mRNA	Protein	Activity	Proliferation	Differentiation	Maturation
Molecular factors						
DNA	Methylation ↓ [137, 138, 140, 170]	GR-KO ↓ [171]	NA	NA	NA	GR-KO ↓ [172]
miRNA	miR-124 ↓ [103, 173]	↓	miR-124 ↓ [103, 173]	miR-124 ↑ [105]	miR-124 ↑	Nd
GR Co-regulators	NA	NA	DCL ↑ [136] Ube3a ↑ [134]	SMO ↑ [174] DKK1 ↓ [175] Ube3a = [133]	Ube3a = [133]	Ube3a ↓ [133]
Genes with GREs	NA	NA	CALD1 ↑ [109] SGK1 ↑ [130] Npas4 ↓ [176]	SGK1 ↓ [130] Npas4 = [177]	CALD1 ↓ [109]	Npas4 ↓ [177]
GR siRNA	GR ↓ [108]	NA		GR = [108]	GR ↑ [108]	GR ↑ [108]
CORT	NA	NA	CORT injection ↑ [64, 178, 179] Adrenal ectomy ↓ [14, 64]	CORT injection ↓ [178] Adrenal ectomy ↑ [14]	CORT injection ↓ [179]	CORT injection ↓ [64] Adrenal ectomy ↑ [64]
Environmental factors						
Early life	Maternal care ↑ [180]	NA	Prenatal stress ↑ [181–183] Maternal care ↑ [180]	Prenatal stress ↓ [181, 183] Maternal care = [184] Maternal deprivation ↓ [141]	Maternal deprivation ↓ [141]	Prenatal stress ↓ [182]& = [181] Maternal care ↑ [184] Maternal deprivation = [141]
Aging	NA	↓ [92, 146, 147]	↑ [185, 186]	↓ [143]	↓ [145, 187]	
Social interaction	NA	NA	Social housing ↑ [188] Isolation ↓ [69] Defeat ↑ [189]	Social housing ↑ [188] Isolation ↓ [69, 190] Defeat ↓ [61–63, 191]	Defeat ↓ [192]	Defeat ↓ [192] Social avoidance ↑ [63] Communal nesting ↑ [193]
Physical activity	NA	NA	Running ↑ [69] Running = [66, 194]	Running ↑ [69] Stressed = [194]	Running ↑ [69]	Running ↑ [195]
Stress	NA	NA	Acute ↑ [99] Chronic ↑ [196]	Acute ↓ [99, 197, 198] Chronic ↓ [199–201]	Acute ↓ [197, 198] Chronic ↓ [200]	Chronic = [199] Chronic ↓ [200]

The altered morphology and mis-positioning of newborn granule cells with reduced GR expression raise the question about the consequences for hippocampal functioning. Contextual fear conditioning, a task that is often used to monitor functional consequences of altered adult neurogenesis [110, 111], showed impairment of contextual but not of cued freezing behavior in mice with reduced GR expression in newborn, 5-week-old granule cells [108]. This behavioral finding is remarkable, as only a limited number of cells, approximately 20,000, are transduced by the lentivirus [107], which further underscores the importance of adult neurogenesis for proper hippocampal functioning.

**Fig. 4:**
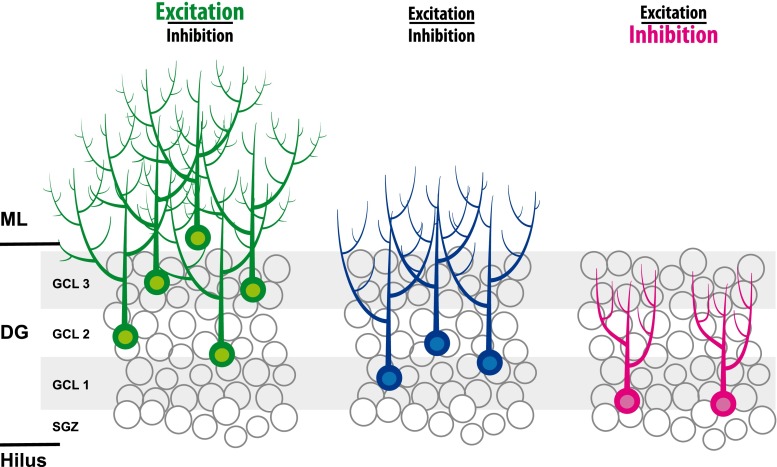
The excitation–inhibition balance determines the number and final positioning of newborn granule cells. Excessive excitation, as is the case after seizures, stroke, and GR knockdown, leads to an increase and ectopic location of newborn granule cells. Reversely, excessive inhibition leads to reduced numbers of newborn cells. Although suggested in this cartoon, it is unknown whether newborn granule cells are ectopically located in the GCL. For further details, see main text

Aberrant placement of newborn granule cells has also been found in other disease-related models. Neuronal diseases, characterized by extensive glutamate release, such as epilepsy and stroke, are associated with massive neurogenesis [112, 113], dendritic abnormalities, and ectopic positioning of newborn cells [114]. Remarkably, in the case of status epilepticus-induced changes in neurogenesis, newly formed neurons are found at ectopic locations [115]. Similarly, as after GR reduction, these ectopic adult-born neurons are hyper-excitable, and are believed to be part of the mechanisms underlying increased susceptibility towards seizures (see Fig. 4) [29, 116, 117]. Reduced levels of disrupted-in-schizophrenia-1 (DISC1) in newborn granule cells cause a highly similar phenotype as after reduced GR levels. DISC1 knockdown in NPCs exhibit accelerated neuronal differentiation, functional integration at ectopic locations and impairment in hippocampus-dependent memory consolidation [118, 119]. Collectively, these studies suggest that integration of newborn granule cells at ectopic locations may contribute to the pathogenesis of both neurological and psychiatric diseases and possibly are part of mechanisms underlying chronification of paroxysmal disorders such as stroke, epilepsy, and schizophrenia [117]. As the GR has been implicated in epilepsy and schizophrenia [120–126], aberrant GR signaling might be part of the mechanisms underlying aberrant integration of newborn cells in these diseases.

The studies outlined above suggest that the level of GR expression is important for adequate adult neurogenesis and should be in a narrow window. Too low expression leads to ectopic integration of newborn granule cells and too high expression may block proliferation and differentiation of NPCs. In line with the importance of a narrow window for GR expression is the finding that a 30 % reduction of GR protein levels results in a 70 % reduction of the expression of glucocorticoid-induced leucine zipper (GILZ), a GR-responsive gene [103]. Indeed, intensive research of the last decades has revealed a myriad of mechanisms all aiming at the regulation of GR activity (see Table 1). Alternative splicing of the GR gene results in at least three different GR isoforms. In addition, alternative translation initiation results in the formation of another eight different GR isoforms. Each of these GR isoforms is expressed in a cell-specific way and control the expression of a unique set of genes [127, 128]. As such, these GR isoforms may contribute to mechanisms underlying cell-specific responses to CORT. GR activity can also be modulated by GR phosphorylation [129]. In human neuronal progenitor cells, GR responsiveness towards CORT also depends on GR phosphorylation by serum- and glucocorticoid-inducible kinase 1 phosphorylation, a target gene for activated GRs [130]. A further complicating factor for the outcome of GR action is the interaction with other proteins, in particular co-activators and co-inhibitors that are expressed in a cell-specific manner in the brain (for review, see [131]). Relevant for adult neurogenesis is Ube3a, a co-activator of the GR, which is crucially involved in the Angelman syndrome [132]. Lack of Ube3a expression not only leads to impaired cognition and decreased numbers of NeuN/BrdU-positive cells [133] but may also result in impaired GR signaling [134]. CBP is another important cofactor and integration point, bridging CREB or zif68/egr1 activity with GR signaling [131, 135]. Together with the regulation of GR levels by neurogenesis-related microRNA-124 [103] and the presence of specific retrograde transport mechanisms for the GR in neuronal progenitor cells such as DCL [136], the different mechanisms outlined above clearly indicate that GR expression and activity are under tight control in neuronal progenitor cells.

An important question that emerges is what type of (environmental) factors regulating adult neurogenesis also affect GR levels in neuronal progenitor cells (see Table 1). Interestingly, early life events, such as maternal separation and parental care, are known to reduce GR levels at adult age by epigenetic programming of the GR promoter [137–140] and is also associated with impaired adult neurogenesis [141, 142]. Aging is associated with lower rates of hippocampal neurogenesis [143–145], impaired negative feedback of CORT on the HPA axis, and reduced levels of the GR [146, 147]. Chronic stress is another factor negatively affecting both GR levels and adult neurogenesis [61, 148–150]. It is unknown whether aging, early life events, and chronic stress directly affect GR levels, thereby impairing proliferation and differentiation of neuronal progenitor cells. It is likely, however, that these effects are indirect, as proliferation, differentiation, migration, and survival depend on neurotrophins and excitatory input, first by GABA input and later in development by glutamatergic input. In other words, different phases of adult neurogenesis require excitatory neuronal activity [for review, see 151]. Chronic stress not only reduces GR levels but also impairs glutamate release via BDNF [152] and reduces expression of other neurotrophins as well [86, 153]. Thus, it seems likely that environmental factors negatively affecting adult neurogenesis attenuate multiple excitatory and inhibitory signaling cascades through the GR.

## The glucocorticoid receptor and the excitation-inhibition balance in neuronal progenitor cells

**Fig. 5:**
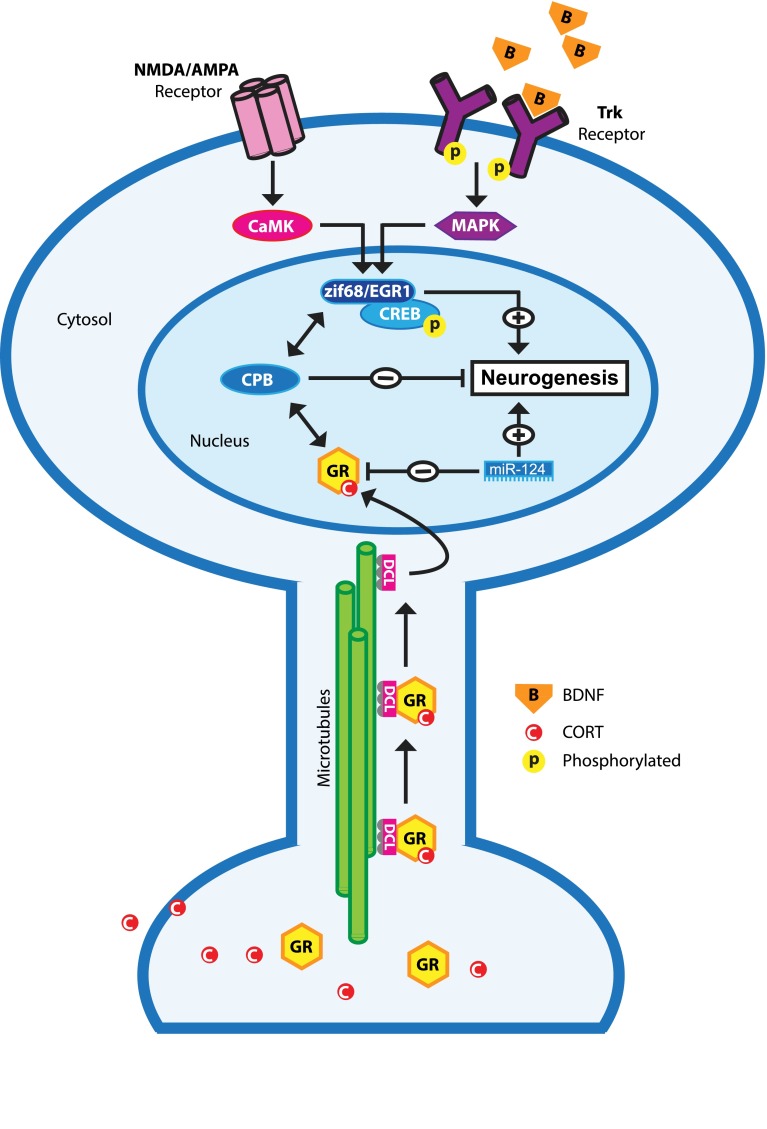
Hypothetical model illustrating possible molecular mechanisms underlying the action of the glucocorticoid receptor in the excitation-inhibition balance in NPCs. Activation of NMDA and AMPA receptors lead to an intracellular signaling cascade leading to the activation of the pro-neurogenic transcription factors CREB and zif68/EGR via CaMK. Likewise, binding of neurotrophins (e.g., BDNF) to Trks leads to activation of CREB and zif68/egr1 via the MAPK pathway. CORT-activated GR is retrograde transported by doublecortin-like (DCL) along the microtubules towards the nucleus. GR may interact with p-CREB by interaction with co-factors such as CREB binding protein (CPB). The net result of this GR-CPB-CREB interaction is the decreased CREB activity and subsequent inhibition of neurogenesis. Pro-neurogenic miR-124 represses GR activity, which may facilitate neuronal differentiation via NMDAR and/or Trk activation

The precise mechanisms by which GR levels directly regulate proliferation and neuronal differentiation in neuronal progenitor cells are unknown. As a transcription factor, the GR affects the expression of hundreds of genes in the hippocampus [154, 155] and without a doubt a number of these genes are involved in the differentiation of neuronal progenitor cells. We suggest that the majority of these GR-responsive genes are regulated by a transrepression model (see also Fig. 5). Studies aiming at the identification of GR-responsive genes in the hippocampus showed that 1 h after corticosterone exposure all responsive genes are downregulated, suggesting a GR-mediated transrepression effect of fast glucocorticoid action [156]. Also, a GR-mediated transactivation mechanism seems to underlie retarded migration of immature neurons in the developing rat brain [109]. This finding strongly suggests that proliferation and neuronal differentiation of neuronal progenitor cells require reduced GR activation, which seems more in line with a transrepression model. Also, this finding is in line with the repression of GR translation of the proneurogenic microRNA-124 [103]. However, recent in vitro studies indicate involvement of a GR-mediated transactivation effect in the pro-neurogenic action of antidepressants [157], which suggest that the GR function in neuronal progenitor cells is context dependent, for example depending on serotonin signaling.

Proper proliferation and neuronal differentiation of neuronal progenitor cells require excitatory input. As outlined before, in early stages this is excitatory GABA [41] and later this is glutamate that act on AMPA receptors and NMDA receptors [158, 159]. Another group of molecules that stimulate neurogenesis are neurotrophins, such as BDNF and VEGF, which are induced and released by environmental enrichment and learning, thereby promoting neurogenesis. Neurotrophins bind to tyrosine kinase receptors (Trk), membrane-bound receptors that are necessary for adult neurogenesis [160]. Activated AMPA receptors, NMDA receptors, and Trk receptors are all known to activate an intracellular signaling cascade that results in the activation of transcription factors that subsequently stimulate proliferation of neuronal precursor cells, their differentiation, and functional integration in hippocampal circuits (for review, see [161]). Examples of such transcription factors are Zif68/egr1 [162] and CREB [163–165], which are key transcription factors in the development and survival of newborn granule cells. Activated GR may interact with these pathways either by binding directly to these transcription factors and/or by interaction with co-factors such as CBP [166] (for review, see [131]). The net result of this GR–CPB–CREB interaction is the decreased CREB activity. The emerging picture in this concept is that CORT and the activated GR dampen excitatory input in neuronal progenitor cells thereby preventing the neurogenesis process from overshoot. Reduction of GR levels, e.g., by miR-124 (see Fig. 5) in NPCs or removal of CORT by adrenalectomy will relief this functional brake on excitatory signaling leading to increased proliferation, differentiation and (aberrant) integration of newborn granule cells in hippocampal circuits. Extrapolation of this model may explain ectopic newborn granule cells in the hilus after seizures, which are characterized by excessive glutamate and neurotrophin release, in particular BDNF. Other paroxysmal disorders like migraine are often also explained by a disturbed excitation-inhibition balance [167] with high levels of cortical glutamate release, which may reach limbic structures during cortical spreading depression [168] and increases proliferative activity in the dentate gyrus [112]. Reversely, chronic high levels of CORT and diminished neuronal activity, i.e., diminished glutamate signaling and neurotrophin release—often associated with psychiatric diseases like depression—may lead to reduced proliferation, differentiation and survival of newborn granule cells (see Fig. 4) [27, 34, 89]. However, it is presently unknown if this high CORT-low glutamate signaling will lead to diminished neuronal migration and functional integration of newborn granule cells mainly in the inner layer of the GCL.

## Perspectives

During the last decade, tremendous progress has been made in understanding adult neurogenesis. The process of morphological changes of neuronal stem cells leading to mature excitatory granule cells, the synaptic connectivity, functional integration and, to a lesser extent, the functional implications for learning and cognition, has been described in considerable detail. Similarly, many genes that are orchestrating the different stages of adult neurogenesis have been identified and numerous intrinsic and extrinsic factors influencing the rate of proliferation and fate of resulting newborn granule cells have been documented. Strangely, despite the fact that stress and stress hormones were one of the earliest discovered factors that manipulate adult neurogenesis [14, 64], their action on neurogenesis is still not fully understood. Likely, this is due to the complexity of the stress system that is controlled by several brain areas and peripheral tissues, i.e., the HPA axis; to different physiological responses after acute stress versus chronic stress and as outlined above, to the complex control of the GR, the main mediator of the stress response in relation to adult neurogenesis. A number of issues remain to be resolved. For example, which GR isoforms are expressed in neuronal progenitor cells. Perhaps more importantly, can reduced GR levels, as observed after chronic stress, aging, and traumatic early life events, also be found in NPCs? Does epigenetic programming of the GR promoter by early life stress also take place in NPCs? If so, what will be the consequences for adult neurogenesis with respect to the positioning of newborn granule cells?

Both chronic stress, a dysfunctional HPA axis, and aberrant neurogenesis have been implicated in psychiatric diseases [27, 33]. A proper excitation-inhibition balance seems key for the control of both the stress system and adult neurogenesis. Both the MR and GR are important mediators for the stress response and interact with proteins that are activated by neuronal activity. Therefore, the question emerges: are these receptors suitable therapeutic targets for restoring excitation-inhibition balances in not only psychiatric diseases [53] but also in paroxysmal disorders like epilepsy [169]? With availability of numerous potent GR and MR antagonists and agonists, which have been developed by the pharmaceutical companies during the last 50 years, and modern genetic technologies like in vivo viral delivery of RNA-interference molecules, these questions can now be addressed.
